# Inhibition of eIF2α Phosphorylation by Peste des Petits Ruminant Virus Phosphoprotein Facilitates Viral Replication

**DOI:** 10.3389/fvets.2021.645571

**Published:** 2021-07-06

**Authors:** Niyokwishimira Alfred, Bang Qian, Xiaodong Qin, Xiangping Yin, Meera Prajapati, Yongxi Dou, Yanmin Li, Zhidong Zhang

**Affiliations:** ^1^State Key Laboratory of Veterinary Etiological Biology, Lanzhou Veterinary Research Institute, Chinese Academy of Agricultural Sciences, Lanzhou, China; ^2^College of Animal Husbandry and Veterinary Medicine, Southwest Minzu University, Chengdu, China

**Keywords:** peste des petits ruminants virus, dephosphorylation, eIF2α, GADD34, phosphoprotein

## Abstract

Peste des petits ruminant virus (PPRV) causes a highly contagious disease in small ruminants. The molecular mechanism of PPRV replication and its interactions with hosts are poorly studied. In other paramyxoviruses, the viral phosphoprotein (P) has been associated with multiple functions for key biological processes such as the regulation of transcription, translation, and the control of cell cycle. Phosphorylation of the α subunit of eukaryotic initiation factor 2 (eIF2α) is an important process for gene regulation in host cells under stress, including viral infection. In the present study, molecular mechanisms associated with PPRV replication and viral interaction with host cells were investigated. We describe the ability of PPRV to dephosphorylate eIF2α and the potential of PPRV P protein to induce the host cellular growth arrest DNA damage protein (GADD34), which is known to be associated with eIF2α dephosphorylation. Furthermore, we observed that PPRV P protein alone could block PERK/eIF2α phosphorylation. We speculate that PPRV exploits eIF2α dephosphorylation to facilitate viral replication and that PPRV P protein is involved in this molecular mechanism. This work provides new insights into further understanding PPRV pathobiology and its viral/host interactions.

## Introduction

Peste des petits ruminants (PPR) is a highly contagious disease of both domestic and wild small ruminants. The morbidity and mortality rates of the disease can reach up to 100% in susceptible animals, leading to important economic losses (1–3). The genome of PPR virus (PPRV), which belongs to genus *Morbillivirus*, family *Paramyxoviridae*, is organized into six structural protein-encoding genes and two non-structural protein-encoding genes arranged in the order of 3'-N-P/C/V-M-F-HN-L-5' (4–6). The two non-structural proteins (C, V) are translated from the open reading frame (ORF) of the phosphoprotein (P), which have been associated with viral replication, pathogenesis, and immunity in a yet unclear mechanism (4, 7). In other morbilliviruses, the functions of their structural and non-structural proteins have been extensively studied. However, in PPRV, the function of viral proteins has been long speculated based on their function in other morbilliviruses (8). The P protein of PPRV is a translational product of a putative molecular weight of ~60 kilodaltons (kDa) but migrates slowly on sodium dodecyl sulfate–polyacrylamide gel electrophoresis (SDS-PAGE) with an observed molecular weight of 79 kDa in size. In other morbilliviruses, the average of the observed molecular weight of P protein stands between 72 and 86 kDa. This variation in protein sizes has been associated with its acidic nature and post-translational phosphorylation, a property it shares with other proteins with rich threonine and serine (5). In PPRV, the functional importance of the PPRV P protein is not yet fully understood. However, in paramyxoviruses, the P protein is associated with multiple functions including its interaction with the C-terminal of the N protein, which is critical for key biological processes such as the regulation of transcription, translation, and the control of the cell cycle (9). In Rinderpest virus (RPV), P protein is a key determinant of cross-species morbillivirus pathogenicity and a vital element of the viral L-polymerase complex, while its oligomerization, rather than phosphorylation, is required for transcription/replication and the RNA-dependent RNA polymerase (RdRp) complex (10, 11). In morbilliviruses, the expression levels of P, V, and C proteins are likely regulated in the same way and the editing process regulates the levels of P and V proteins. These functions suggest crucial roles for these proteins in facilitating virus replication by downregulating host immunity. However, the functions of these proteins in PPRV replication and pathogenicity have not been fully investigated. Recently, the PPRV V and P proteins have been implicated in the suppression of STAT-mediated interferon signaling (12, 13). It is known that for viruses to complete their replication cycles and viral release from infected cells, some viruses inhibit apoptosis to prevent the premature death of host cells, while others may induce apoptosis to facilitate the release and dissemination of nascent viruses into the neighboring cells to maintain their viral pathogenicity. Apoptosis has been comprehensively described in other members of the family *Paramyxoviridae* (14, 15). In PPRV, autophagy and apoptosis have been briefly described, suggesting their potential role in viral replication and invasion of host defense (16–18). However, the molecular mechanisms behind the regulation of autophagy and apoptosis in PPRV infection remain unclear. Therefore, investigation of the molecular mechanisms and viral proteins involved in the modulation of cell fate during PPRV infection is required (8, 19). Moreover, PPRV is classified among viruses as having a relatively long life cycle (20). Consequently, it is reasonable to hypothesize the existence of a specific mechanism for such viruses to prevent early host cell death in order to complete their life cycle and virus release. Interactions between stressed cellular proteins and viral proteins have been studied in various types of viruses and host cells (21–23). Physiologically, the eukaryotic initiation factor 2 (eIF2α) phosphorylation leads to the initiation of a new reconfiguration of gene expressions to manage cell stress conditions through a reduced global translation (24). However, during viral infection, these cellular stress responses are usually disturbed at various levels to ensure viral survival and normal viral protein translation. This study was designed to investigate the molecular mechanism associated with PPRV replication and its viral/host interaction with a focus on eIF2α phosphorylation. We found that PPRV infection potentially represses PERK/eIF2α phosphorylation in Vero cells. Furthermore, analysis of PPRV P protein revealed a critical role in the modulation of post-translational machinery *via* interaction with cellular regulatory proteins such as pro-apoptotic and pro-survival related proteins. Similarly in PPRV infection, we discovered that PPRV P protein alone could block PERK/eIF2α phosphorylation and induce the upregulation of the growth arrest DNA damage protein (GADD34), a cellular protein that is known to be involved in the dephosphorylation of eIF2α (25). Thus, we speculate that the repression of eIF2α phosphorylation by PPRV P protein *via* induction of cellular GADD34 expression is among the mechanisms conducted by PPRV to regulate long-term survival of host cells, which in turn is beneficial to virus replication.

## Materials and Methods

### Cells, Virus, Antibodies, and Reagents

African green monkey kidney cells (Vero) and human embryonic kidney (HEK293T) were obtained from Lanzhou Veterinary Research Institute (LVRI), Lanzhou, China. All cells were sub-cultured and maintained in Dulbecco's Modified Eagle's medium (DMEM) supplemented with 10% fetal bovine serum (FBS) treated with 1% penicillin–streptomycin (Gibco, Grand Island, NY, USA). Infected or transfected cells were maintained in DMEM supplemented with 2% FBS treated with 1% penicillin–streptomycin. All cells were grown or maintained at 37°C supplied with 5% of CO_2_. A vaccine strain of PPRV Nigeria 75/1 (accession No. X74443) at a titer of 10^4.6^ TCID_50_was obtained from the frozen stock (−80°C) of LVRI, China. During experiments, Vero cells were infected with PPRV at the multiplicity of infection (MOI) = 3, or mock-infected with free culture media and incubated at 37°C supplied with 5% CO_2_ at indicated times according to the experimental designs. At suitable times, PPRV-infected or mock-infected cells were either treated or left untreated, and then harvested and prepared for either real-time quantitative polymerase chain reaction (RT-qPCR) or immunoblotting, or stained for immunofluorescence assays (IFAs). A mouse monoclonal antibody against N-protein of PPRV was obtained from Dr. Xuelian Meng, LVRI, China. The commercial antibodies and reagents used are as follows: Rabbit anti-PERK/EIF2AK3 polyclonal antibody (24390-1-AP), Rabbit anti-EIF2S1 polyclonal antibody (11170-1-AP), Rabbit anti-GADD34 polyclonal antibody (10449-1-AP), Rabbit anti-ATF4 polyclonal antibody (10835-1-AP), Mouse anti-His-Tag monoclonal antibody (66005-1-Ig), Rabbit anti-G3BP1 (13057-2-AP), Rabbit anti-TIA-1 (12133-2-AP), and Mouse anti-β-actin. Monoclonal antibody (60008-1-ig) was purchased from Proteintech; Rabbit anti-phospho-PERK (Thr982) polyclonal antibody (DF7576) was purchased from Affinity Biosciences; Rabbit anti-EIF2S1(phosphoS51) antibody (ab32157), Rabbit anti-mouse IgG H&L (HRP) (ab6728), Goat anti-rabbit IgG H&L (HRP) (ab205718), Rabbit Anti-DDDDK tag antibody (equivalent to FLAG antibodies from Sigma) (ab1162), Mouse anti-ATF6 (ab11909), Rabbit anti-Bip/GRP78 (ab21685), Rabbit anti-GFP (ab6556), Goat anti-rabbit IgG H&L (Alexa Fluor 488) (ab150077), and Goat anti-mouse IgG H&L (Alexa Fluor 647) (ab150115) were purchased from Abcam. Halt Protease and Phosphatase Inhibitor cocktail (78442) and Rabbit anti-GADD34 (PA1-139) polyclonal antibody were purchased from Thermo Fisher Scientific, USA. Rabbit anti-CHOP (D46F1) monoclonal antibody was purchased from Cell Signaling. RIPA cell lysis buffer (P0013B) was purchased from Beyotime Biotech.

### Plasmid Construction

The full-length ORFs of the PPRV P and PPRV N genes were PCR amplified from cDNA synthesized from Vero cells infected with PPRV. A His-tag was added to the C-terminal of the P gene by PCR cloning and ligated *via Mlu*I and *Not*I cloning sites of the pCI mammalian expression vector purchased from Promega (Madison, WI, USA), under the control of T7polymerase promoter to generate a pCI-P His plasmid expressing a His-tagged PPR P protein. The ORF of N genes was inserted by fusion with Flag tag into pCMV-tag 2 mammal expression vector (Clontech) between *Srf* I and *Eco*RI to generate a Flag-pCMV-N protein-expressing Flag-tagged PPRV N protein under the control of the CMV promoter. All plasmid constructs were used to transform DH5α competent cells (Takara). A stock of purified His-tagged pCI-P and Flag-tagged pCMV-N plasmids was aliquoted and kept at −20°C until use. Inserts of plasmid constructs were confirmed by DNA sequencing. A Flag-tagged GADD34 plasmid (pXJ40 GADD34 Wt) (1–674aa) was previously constructed and published (26). Primer sequences used for plasmid construction in this study are listed in Table 1.

**Table 1:** The primers used for plasmid constructions.

**Name**	**Primer sequence (5'-3')**
P-F	CGACGCGTATGGCAGAAGAACAAGCATACCAT
P-R	ATAAGAATGCGGCCGCTTAGTGGTGGTGGTGGTGGTGCGG CTGCTTGGCAAGAATG
**His-tag**	
N-F	TTGGCCCGGGCGGCTACTCTCCTTAAAAGCTTG
N-R	5- CCGGAATTCTTAATCAGCTGAGGAGATCCTTGTCG

### Transfection and Transient Expression of Target Proteins

When HEK293T or Vero cells were grown at 70–80% confluence, the cells were transfected or co-transfected with appropriate amount of either pCI-P His/Flag-pCMV-N or pXJ40 GADD34 plasmids, or mock-transfected with the same amount of empty pCI/ pCMV vector or free transfection reagent as a negative control using Attractene Transfection Reagent (301007) (Qiagen) according to the manufacturer's instructions. At the indicated time, cells were lysed and analyzed by SDS-PAGE followed by Western blotting or stained for IFA analysis.

### Chemical Treatment

When Vero cells were grown at 70–80% confluence, the cells were infected with PPRV at MOI = 3 or mock-infected. After 2-h adsorption, infection media was changed to fresh media treated with either different concentration of Salubrinal (CAS405060-95-9) or 200 nM integrated stress inhibitor (ISRIB) (CAS1597403-47-8) (Santa Cruz Biotechnology, Santa Cruz, CA, USA) at indicated times. Cells were harvested and total RNA was extracted for RT-qPCR or lysed for SDS-PAGE followed by immunoblotting analysis, respectively, for PPRV expression. Vero cells infected with PPRV or mock-infected were incubated and then treated with 2 mM dithiothreitol (DTT) (CAS27565-41-9) (Santa Cruz) or 1 μM Thapsigargin (TG) (HY-13433) (MedChemExpress) for 2 h up to 48 h.p.i. for DTT or 12 h up to 60 h.p.i. for TG, respectively, to induce ER stress. Cells were harvested and lysed for SDS-PAGE analysis followed by immunoblotting of target proteins.

### Immunoblotting

Infected or transfected cells were washed twice with phosphate-buffered saline (PBS) and lysed in appropriate lysis buffer supplemented with a protease and phosphatase inhibitor cocktail according to the manufacturer's instruction and centrifuged at 16,000 × *g* for 10 min. The supernatant was collected and mixed with appropriate protein loading buffer and boiled at 95°C for 5 min. An equal amount of protein was loaded and separated by SDS-PAGE and transferred to polyvinylidene difluoride (PVDF) membrane according to the standard protocols. Target proteins were then probed with specific primary antibodies overnight, followed by incubation for 1 h with an appropriate horseradish peroxidase-conjugated (HRP) secondary antibody at proper dilutions. Corresponding signals bands were detected using Millipore Immobilon Western Chemiluminescent HRP Substrate (WBKLS0100).

### Immunofluorescence Assay

Vero or HEK293T cells grown directly on glassware dishes for 12 h at 70–80% confluence were infected/transfected/ or mock-infected/transfected at indicated times. Cells were washed thrice for 5 min with TBS, fixed with 4% paraformaldehyde (PFA) for 30 min at room temperature, and permeabilized with 0.2% Triton X-100-TBS for 5 min. To avoid non-specific bindings, cells were blocked using 5% BSA-TBS for 1 h at room temperature. A proper dilution of primary antibodies was incubated overnight in 1% BSA-TBST at 4°C followed by three washes and incubation with appropriate secondary antibodies for 45 min at room temperature. Stained cells were mounted with 4,6-Diamidino-2-Phenylindole (DAPI) and scanned with confocal fluorescence microscopy TCS SP8 (Leica Microsystems).

### RT-qPCR Assay for Quantification of PPRV RNA

Viral RNA was extracted from samples using the RNeasy Mini Kit (74104) (Qiagen) according to the manufacturer's instruction. RNA was eluted in nuclease-free water, quantified by Nanovue (GE Life science, USA), aliquoted, and stored at −80°C until use. The RT-qPCR assay used in this study was performed in a one-step RT-qPCR system as previously described (27, 28) with some modifications. Briefly, a single tube of 25-μl reaction was prepared with 12.5 μl of Superscript III/Platinum Taq One-step RT-qPCR reaction mix, 1 μl of Superscript III/Platinum Taq One-step RT-qPCR enzyme mix, 5 pmol Taqman probes, 10 pmol of forward/reverse primers each, and 2 μl of total RNA as a template. Cycling conditions were set as follows: 50°C for 15 min (1 cycle), 95°C for 10 min (1 cycle), and 95°C for 15 s followed by 60°C for 1 min (40 cycles). The reaction was run into the Agilent Technologies Stratagene Mx3005P thermocycler (Life Technologies, USA). The standard RNA used for PPRV quantification in RT-qPCR assay was prepared as previously described (27) using full-length ORF of PPRV N protein cloned into the pCMV-Tag2 vector as described in section Plasmid Construction.

### Data Analysis

All the data are presented as mean ± standard deviation (SD) from three replicates (*n* = 3) of at least two independent experiments. The densitometry quantification of the relative intensity band ratio for targeted proteins/β-actin was determined by ImageJ analysis. Two-way ANOVA or multiple *t*-tests were used for statistical analysis with GraphPad Prism 8.0 software with *P*-value (^*^P < 0.05) considered statistically significant.

## Results

### PPRV Infection Results in Dephosphorylation of eIF2α Concurrent With Inhibition of PERK Phosphorylation in Cells

**Figure 1:**
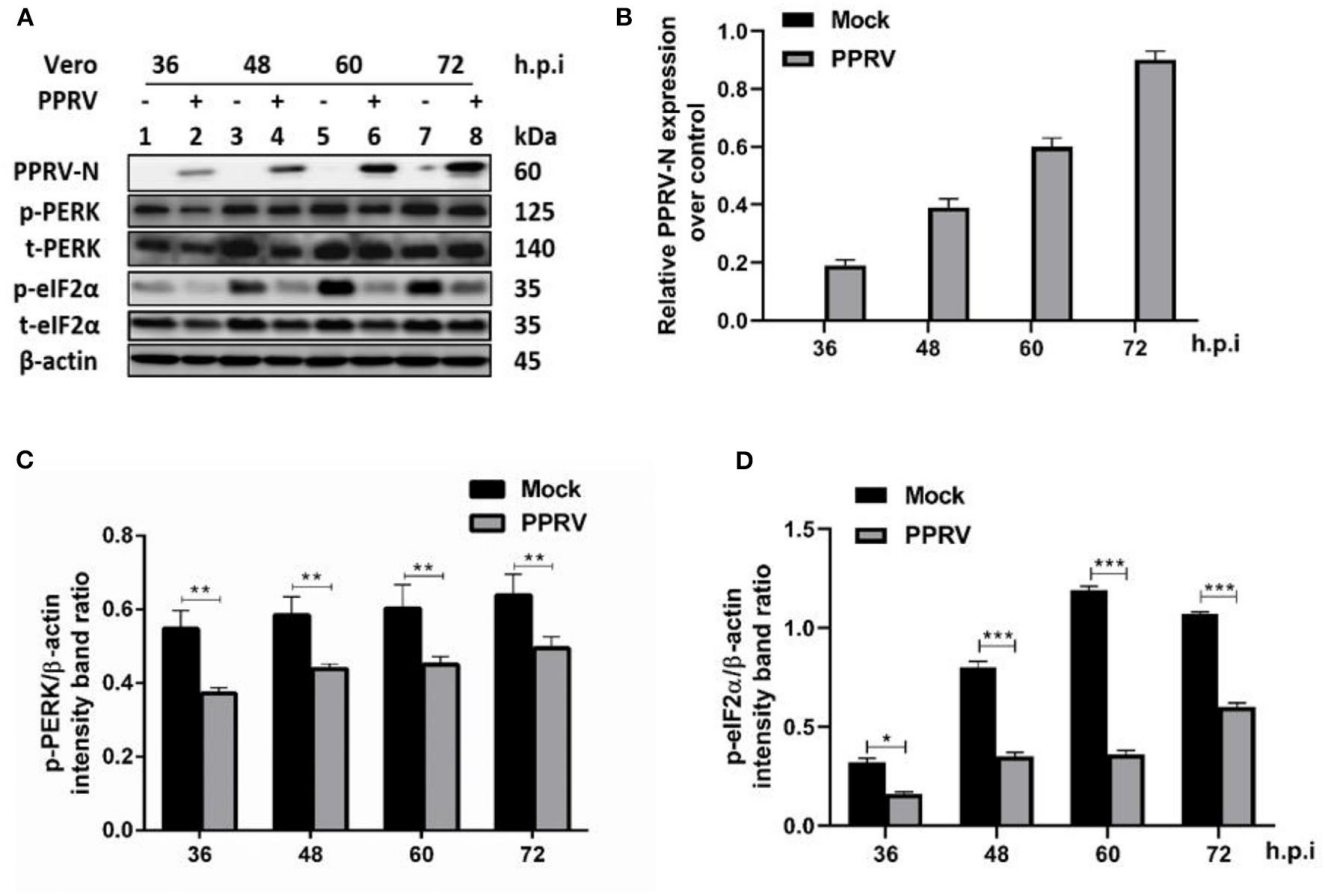
PPRV infection inhibited eIF2α phosphorylation in Vero cells. Vero cells were infected with PPRV or mock-infected. **(A)** The effect of PPRV on target proteins was analyzed by Western blot probed with specific antibodies. **(B)** Relative expression of PPRV in Vero cells over time was analyzed by Western blot probed with a mouse monoclonal anti-PPRV N. **(C,D)** Densitometry quantification of p-PERK and p-eIF2α was normalized to β-actin and fold change was measured within each time point compared with mock-infected by ImageJ analysis. Statistical analysis was performed using GraphPad Prism 8.0 software. Shown are representative immunoblots and the error bars represent the mean ± SD from three independent experiments. The asterisks represent the statistically significant difference between mock and infected cells (*P < 0.5, **P < 0.01, ***P < 0.001).

Several viruses modulate p-eIF2α levels during replication to ensure viral protein synthesis and avoid cellular stress responses. To examine the phosphorylation status of eIF2α in PPRV infection, Vero cells were infected or mock-infected with PPRV at indicated times. Cell lysates were analyzed by SDS-PAGE followed by Western blot for the expression of target proteins. Viral infection was confirmed by Western blot probed with a mouse monoclonal anti-PPRV N antibody (Figures 1A,B). eIF2α and phosphorylated eIF2α (p-eIF2α) at the serine 51 position were analyzed by Western blot stained with specific antibodies. The relative levels of p-eIF2α were normalized to β-actin as an internal loading control protein. Densitometry quantification of p-eIF2α was calculated by ImageJ analysis for 36, 48, 60, and 72 h post-infection (h.p.i.). As shown in Figures 1C,D, the levels of p-eIF2α were significantly lower in infected cells compared to mock-infected cells, indicating that PPRV infection does potentially repress eIF2α phosphorylation. To further investigate the effect of PPRV infection on cellular protein translation, we also examined the expression of the protein kinase R (PKR-like) ER kinase (PERK), which is a well-characterized ER stress-induced translational control pathway (29). PERK and phosphorylated PERK (p-PERK) at Threonine 982 position were analyzed by Western blot stained with specific antibodies. As shown in Figures 1C,D, the results showed a significant downregulation in p-PERK (Figures 1C,D), indicating that PPRV infection does potentially repress both PERK and eIF2α phosphorylation.

### PPRV Infection Results in Upregulation of GADD34 Protein Expression in Cells

**Figure 2:**
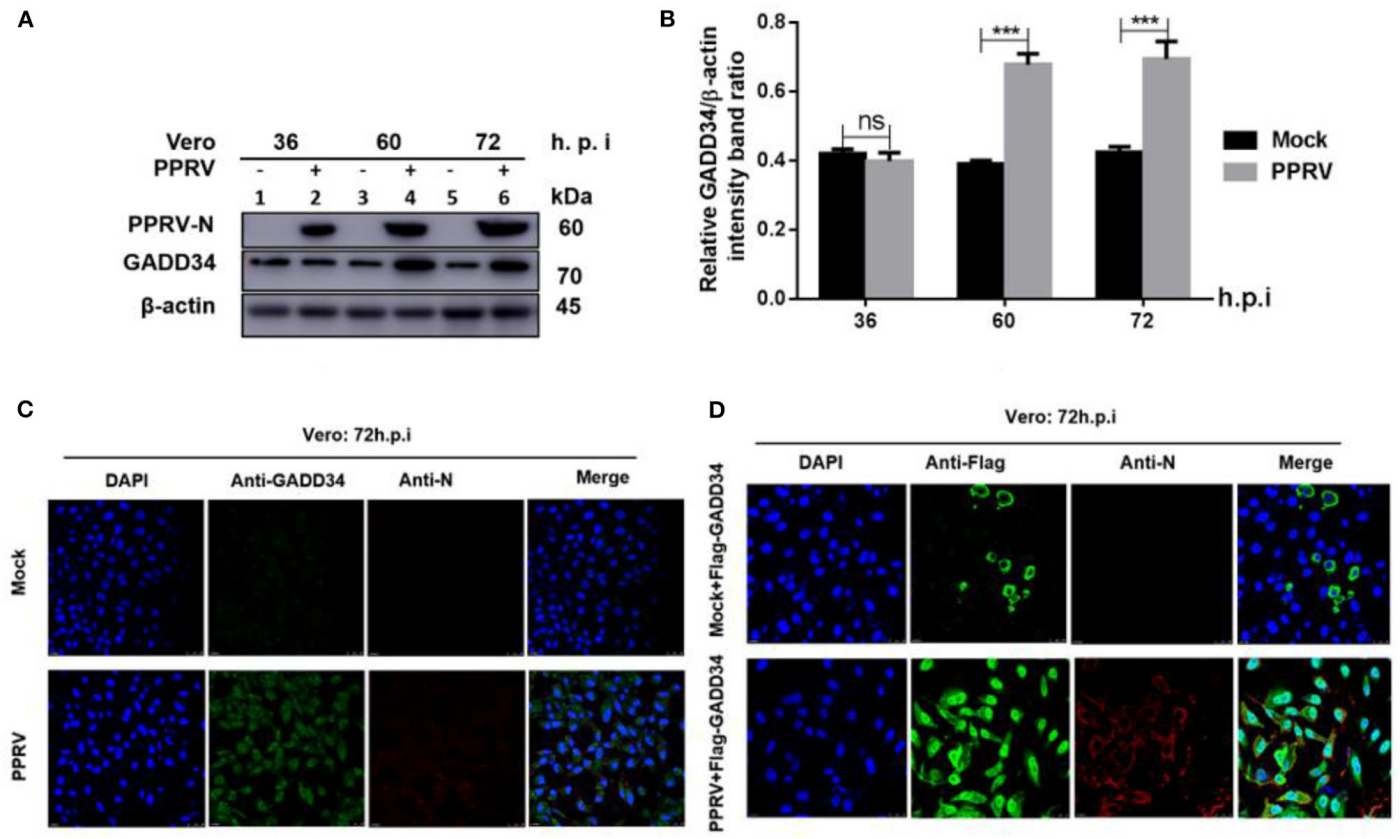
PPRV infection modulates GADD34 protein expression in Vero cells. Vero cells were infected with PPRV or mock-infected at indicated times. **(A)** The effect of PPRV on GADD34 protein expression was analyzed by Western blot. **(B)** Densitometry quantification of GADD34 was normalized to β-actin and fold change was measured within each time point compared with mock-infected by ImageJ analysis. Statistical analysis was performed using GraphPad Prism 8 software. Shown are representative immunoblots and the error bars represent the mean ± SD from three independent experiments. Asterisks represent the statistically significant difference between mock and infected cells (***P < 0.001 and ns, not significant). **(C)** Vero cells were infected with PPRV or mock-infected for 72 h. **(D)** Vero cells were transfected with Flag-GADD34 (12 h) before infection with PPRV or mock infection for a further 72 h. Cells were double-stained with mouse monoclonal antibodies against PPRV N protein (1:50) and Rabbit anti-GADD34 antibodies (PA1-139) 1:100 in **(C)** or Rabbit anti-Flag (ab1162) 1:200 in **(D)** as primary antibodies overnight. Goat anti-rabbit IgG (H&L) Alexa Fluor 488 (ab150077) Green1:500 and Goat anti-mouse IgG H&L (Alexa Fluor 647) (ab150115) Red 1:1000 were used as secondary antibodies for 1 h at room temperature. DAPI was used to stain the cell nuclei (blue).

The molecular mechanism of eIF2α dephosphorylation has been previously described on a feedback inhibition of the unfolded protein response (UPR) by the GADD34 (30). To analyze whether the predicted PPRV-mediated eIF2α repression was GADD34 dependent, new sets of Vero cells were infected with PPRV or mock-infected and then the effect of PPRV on GADD34 expression was analyzed. Whole-cell lysates were prepared and analyzed for 36, 60, and 72 h.p.i. by Western blot. As expected, the level of GADD34 was significantly higher after 36 h.p.i. in PPRV-infected compared to mock-infected cells (Figures 2A,B). Analysis by IFA also showed higher GADD34 fluorescence in PPRV-infected Vero cells than in mock-infected cells (Figure 2C). Reciprocally, to analyze the effect of GADD34 on PPRV replication, another set of Vero cells were transiently transfected with a construct expressing Flag-GADD34 plasmid (26) or mock-transfected with a free transfection reagent before infection with PPRV or mock infection and subjected to IFA analysis. In Vero cells transiently expressing Flag-GADD34 before infection (12 h), the fluorescence of Flag-GADD34 was highly enhanced compared to that of mock-infected cells (see Figures 2C,D). It was interesting to note that in cells overexpressing GADD34 prior to infection, this protein was more concentrated in both the cytoplasm and nucleus under the effect of PPRV infection (Figure 2D). These results suggest a strong reciprocal interaction between PPRV replication and GADD34 expression, which may be linked to the aforementioned PPRV-mediated eIF2α dephosphorylation.

### PPRV Infection Represses eIF2α Phosphorylation Triggered by ER Stress

**Figure 3:**
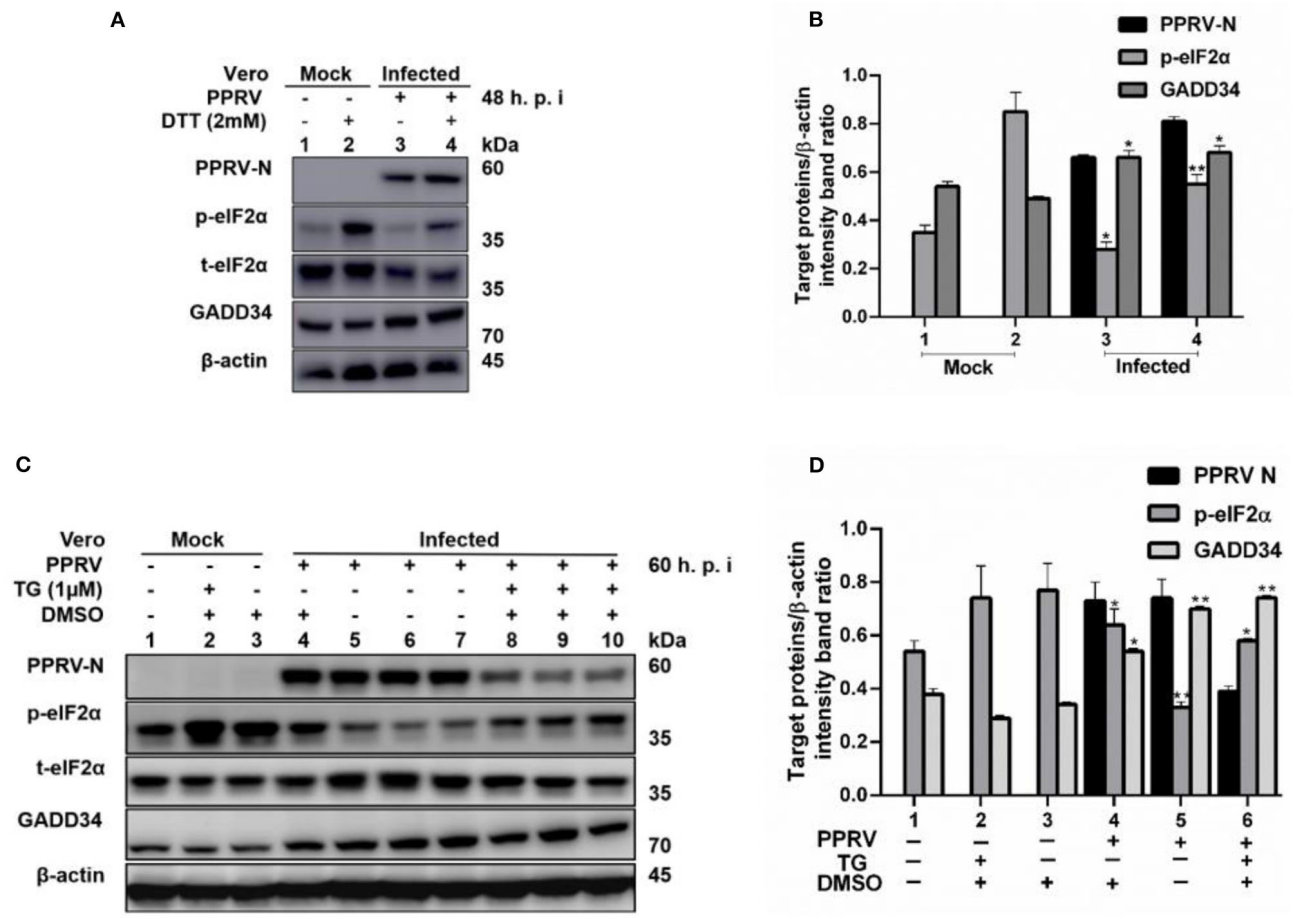
PPRV infection represses ER stress-induced eIF2α phosphorylation in Vero cells. Vero cells were infected with PPRV or mock-infected. **(A,C)** PPRV infection represses eIF2α phosphorylation, induced by DTT and TG, respectively. PPRV-infected or mock-infected cells were treated with DTT (2 mM) or TG (1 μM) for 2 h (for DTT) or 12 h (for TG), respectively, to induce ER stress. The effect of PPRV on p-eIF2α and GADD34 expression in DTT- or TG-treated groups were analyzed by Western blot. **(B,D)** Densitometry quantification of p-eIF2α and GADD34 was normalized to β-actin. Fold change was measured and compared with mock-infected treated or untreated by ImageJ analysis. Statistical analysis was performed using GraphPad Prism 8.0 software. Shown are representative immunoblots with the error bars representing the mean ± SD from three independent experiments. Asterisks represent statistically significant differences between treated and untreated groups (*P < 0.05; **P < 0.01). **#** In the graphic **(D)**, plot 5 represents one set of untreated samples (lines 5 to 7) and plot 6 represents one set of TG-treated samples (lines 8 to 10).

To further confirm that PPRV was modulating eIF2α phosphorylation, Vero cells were infected with PPRV or mock-infected followed by ER stress induction through treatment with either 2 mM DTT for 2 h to a total of 48 h.p.i. or 1 μM TG for 12 h to a total of 60 h.p.i. DTT is a strong ER stress inducer that can induce ER stress in a very short time, while TG is a SERCA-pump inhibitor that inhibits calcium-dependent chaperones, therefore increasing protein misfolding. Both chemicals are known to cause ER stress in several cell lines (31). Cell lysates were analyzed for p-eIF2α (S51), while β-actin was used as an internal control. The results showed that the level of p-eIF2α was very high in all mock-infected cells treated either with DTT or TG, but significantly decreased in PPRV-infected cells treated with DTT or TG. Likewise, neither DTT nor TG could alter the levels of GADD34 in the presence or absence of PPRV (Figures 3A–D). The level of p-eIF2α was significantly more repressed in PPRV-infected groups treated with DTT (Figure 3A, line 4) than in mock-infected treated with DTT (Figure 3A, line 2). In contrast, GADD34 was more upregulated in all PPRV-infected cells treated or untreated with DTT (Figure 3A, lines 3 and 4) compared to mock-infected cells (Figure 3A, lines 1 and 2). In PPRV-infected and TG-treated groups, the level of p-eIF2α was more repressed (Figure 3C, lines 8–10) compared to mock-infected groups treated in either the same condition (Figure 3C, lines 1–3) or with DMSO alone (Figure 3C, line 4). GADD34 was more upregulated in PPRV-infected groups treated either with or without TG (Figure 3C, lines 4–10) compared to mock-infected groups (Figure 3C, lines 1–3). These results support the previous observations that PPRV infection represses eIF2α phosphorylation and potentially induces GADD34. Additionally, the results demonstrate the ability of PPRV, to an extent, to evenly repress the chemically triggered ER stress-mediated eIF2α phosphorylation. Interestingly, cells treated with TG exhibited a reduced expression of PPRV N protein, therefore suggesting a potential ability of TG to inhibit PPRV replication. However, the reduced expression of PPRV N protein following the treatment with TG did not completely suppress its ability to repress eIF2α phosphorylation (Figure 3C, lines 8–10). These results provide evidence that PPRV can dephosphorylate eIF2α, even in the presence of some ER stressors such as DTT and TG.

### Inhibition of eIF2α Phosphorylation Is Beneficial to PPRV Replication in Cells

**Figure 4:**
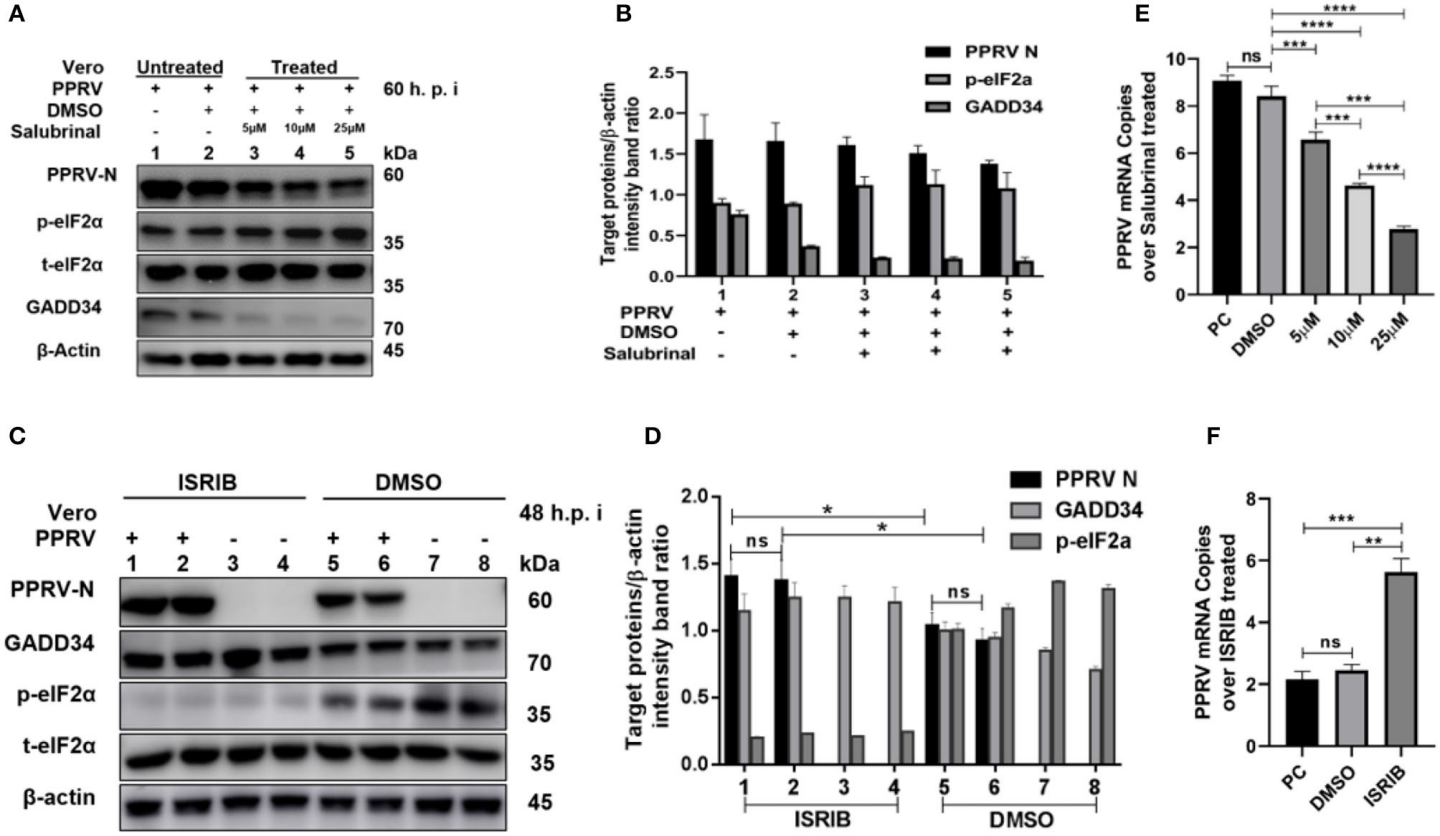
Inhibition of eIF2α dephosphorylation is beneficial to PPRV replication. Vero cells were infected with PPRV or mock-infected. **(A,C)** After 2-h adsorption, infection media was replaced by fresh media treated with 5, 10, and 25 μM of Salubrinal in **(A)** or ISRIB (200 nM) in **(C)** and incubated up to 60 h.p.i. for Salubrinal and 48 h.p.i. for ISRIB. Treatment with DMSO alone was used as control. The effect of PPRV on p-eIF2α in treated or untreated groups were analyzed by Western blot. **(B,D)** Densitometry quantification of p-eIF2α, GADD34, and PPRV-N was normalized to β-actin and fold change between treated and untreated was determined by ImageJ analysis. Statistical analysis was performed using GraphPad Prism 8.0 software. On the graphs, shown are representative immunoblots with the error bars representing the mean ± SD from three independent experiments. **(E,F)** RT-qPCR was used to quantify the mRNA levels of PPRV in samples treated or untreated with Salubrinal **(E)** or ISRIB **(F)**. Viral RNA of the positive control (PC) or vehicle treated with DMSO were compared to that of Salubrinal or ISRIB treated groups. The mean ± standard deviation (SD) was obtained in triplicate (*n* = 3). Statistical analysis was performed using GraphPad Prism 8.0 software using multiple *t*-tests. Asterisks represent statistically significant differences between treated and untreated groups (*P < 0.05; **P < 0.01; ***P < 0.001; ****P < 0.0001; ns, not significant).

To further investigate the role of eIF2α dephosphorylation in PPRV replication, two chemicals with known agonist/antagonist effects on the eIF2α phosphorylation were used to analyze PPRV replication *in vitro*: (1) Salubrinal is a selective inhibitor of eIF2α dephosphorylation (32, 33), and (2) integrated stress inhibitor (ISRIB) is known to reverse eIF2α phosphorylation and the restoration of cellular translation (34). Vero cells were infected with PPRV or mock-infected. After 2-h adsorption, infection media was changed to fresh media treated with different concentrations (5, 10, and 25 μM) of Salubrinal, followed by incubation up to 60 h.p.i. as described in the previous study (35). On the other hand, PPRV-infected or mock-infected cells were treated with 200 nM ISRIB, as described in the previous study (34), followed by incubation up to 48 h.p.i. Both treated and untreated samples were analyzed by RT-qPCR, as described in section Materials and Methods, or loaded on SDS-PAGE followed by probing with a monoclonal antibody against PPRV N protein to examine the expression of PPRV N protein after treatment. As shown in Figure 4A, there was a non-significant difference in expression of PPRV N protein in untreated cells (Figure 4A, line 1) and cells treated with DMSO alone (Figure 4A, line 2). In contrast, the relative expression of PPRV N protein decreased in a dose-dependent manner in cells treated with different concentrations of Salubrinal (Figure 4A, lines 3–5). Interestingly, following the treatment with Salubrinal, the level of PPRV N protein expression was reduced in correlation with the inhibition of GADD34 (Figure 4A, lines 3–5). Conversely, the expression of PPRV N protein was significantly reduced in cells treated with DMSO alone (Figure 4C, lines 5 and 6) when compared to cells treated with ISRIB (Figure 4C, lines 1 and 2) at 48 h.p.i. Moreover, in cells treated with ISRIB, the level of p-eIF2α was significantly decreased while the level of GADD34 significantly increased (Figure 4C, lines 1–4). At the mRNA level, similar results were observed for Salubrinal or ISRIB-treated cells analyzed by RT-qPCR, as shown in Figures 4E,F, respectively. The effect of ISRIB was more significant at the mRNA level compared to the protein expression level, as can be seen in Figures 4D,F. These data indicate that the dephosphorylation of eIF2α is beneficial to PPRV replication.

### Viral P Protein Is Involved in Dephosphorylation of eIF2α During PPRV Infection

**Figure 5:**
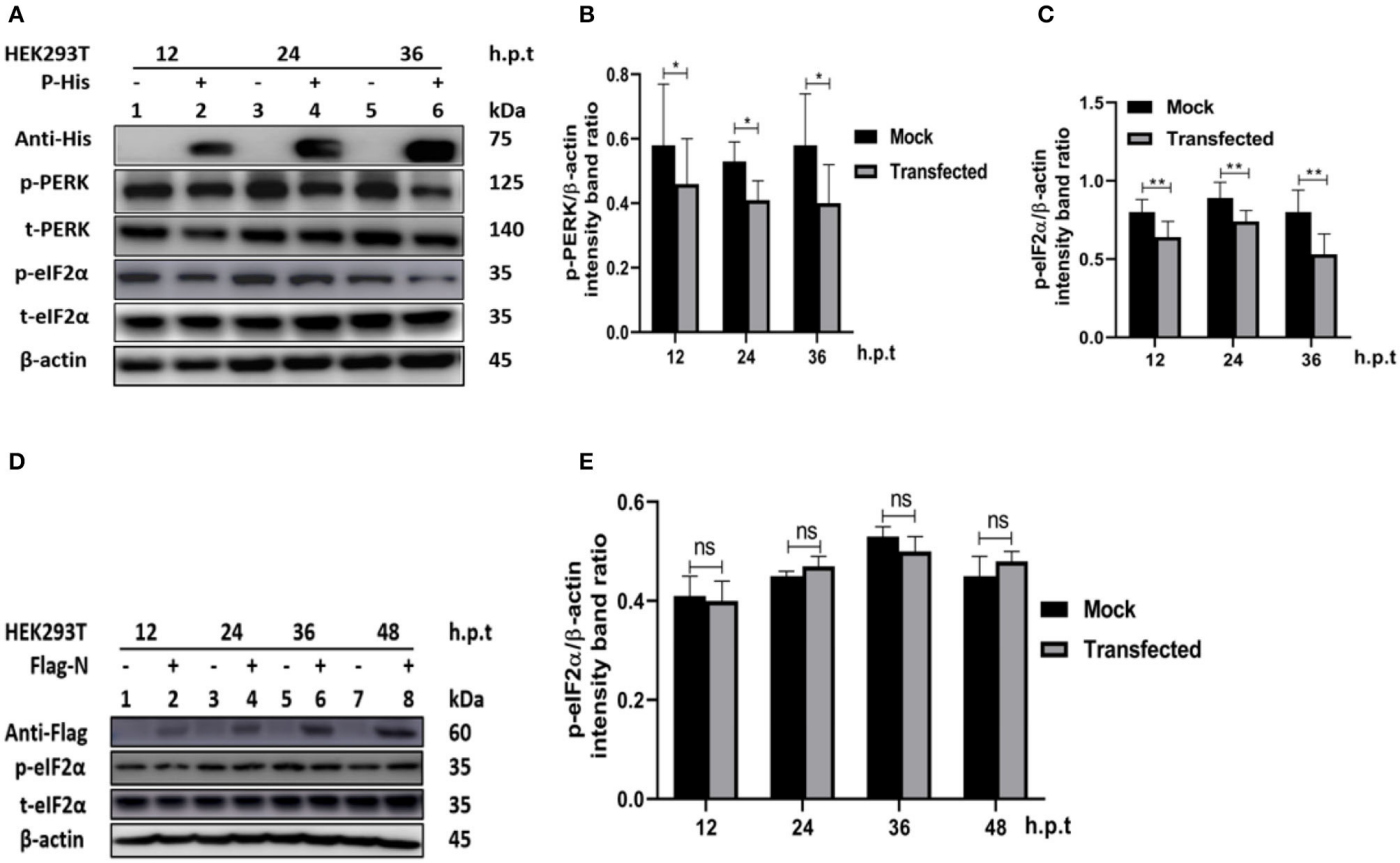
PPRV P protein but not PPRV N protein blocks PERK/eIF2α phosphorylation. **(A–C)** PPRV P protein blocks eIF2α phosphorylation. HEK293T cells were transfected with His-tagged P protein expressing plasmid or mock-transfected with an empty vector at indicated times. The effect of PPRV P protein on p-PERK and p-eIF2α expression was analyzed by Western blot. **(D,E)** PPRV N does not block eIF2α phosphorylation. HEK 293T cells were transfected with Flag-tagged N protein expressing plasmid or mock-transfected with an empty vector at indicated times. The effect of PPRV N on p-eIF2α expression was analyzed by Western blot. In all sets, the expression of PPRV P and PPRV N proteins was confirmed by Western blotting probed with anti-His **(A)** and anti-Flag **(D)** antibodies, respectively. Densitometry quantification of p-PERK **(B)** or p-eIF2α **(C,E)** was normalized to β-actin and fold change was measured and compared with mock-transfected cells within each time point by ImageJ. Statistical analysis was performed using GraphPad Prism 8.0 software. On the graphs, shown are representative immunoblots with the error bars representing the mean ± SD from three independent experiments. Asterisks represent statistically significant differences between mock and transfected cells (*P < 0.5; **P < 0.01; ns, not significant).

In other paramyxoviruses, the P protein performs multiple functions. For example, in RPV, the P protein was shown to be required for replication/transcription (36), whereas in measles virus (MV), the interaction of P-N is required for biological processes such as transcription and regulation of protein translation, and controlling the cell cycle (9). In PPRV, the role of the P protein is not fully understood. To investigate the role of PPRV P protein in PPRV-mediated PERK/eIF2α repressions, HEK 293T cells were transfected with a His-tagged PPRV P protein-expressing plasmid or mock-transfected with an empty vector. The levels of p-PERK and p-eIF2α expression were then analyzed in comparison with mock-transfected cells. The results showed that the PPRV P protein alone was able to repress PERK/eIF2α phosphorylation in HEK293T cells (Figures 5A–C). To further investigate whether PPRV P protein, but not PPRV N protein could induce p-eIF2α downregulation, a Flag-tagged PPRV N protein expressing plasmid was used to transfect HEK293T cells and analyzed for the expression of p-eIF2α at indicated times. Results show that the level of p-eIF2α in cells transfected with PPRV N protein did not exhibit any significant p-eIF2α downregulation (Figures 5D,E). These results indicate that the PPRV P protein, but not the PPRV N protein, is at least one factor involved in PPRV-mediated eIF2α dephosphorylation.

### PPRV P Protein Is Involved in Upregulation of Cellular GADD34 Protein Expression

**Figure 6:**
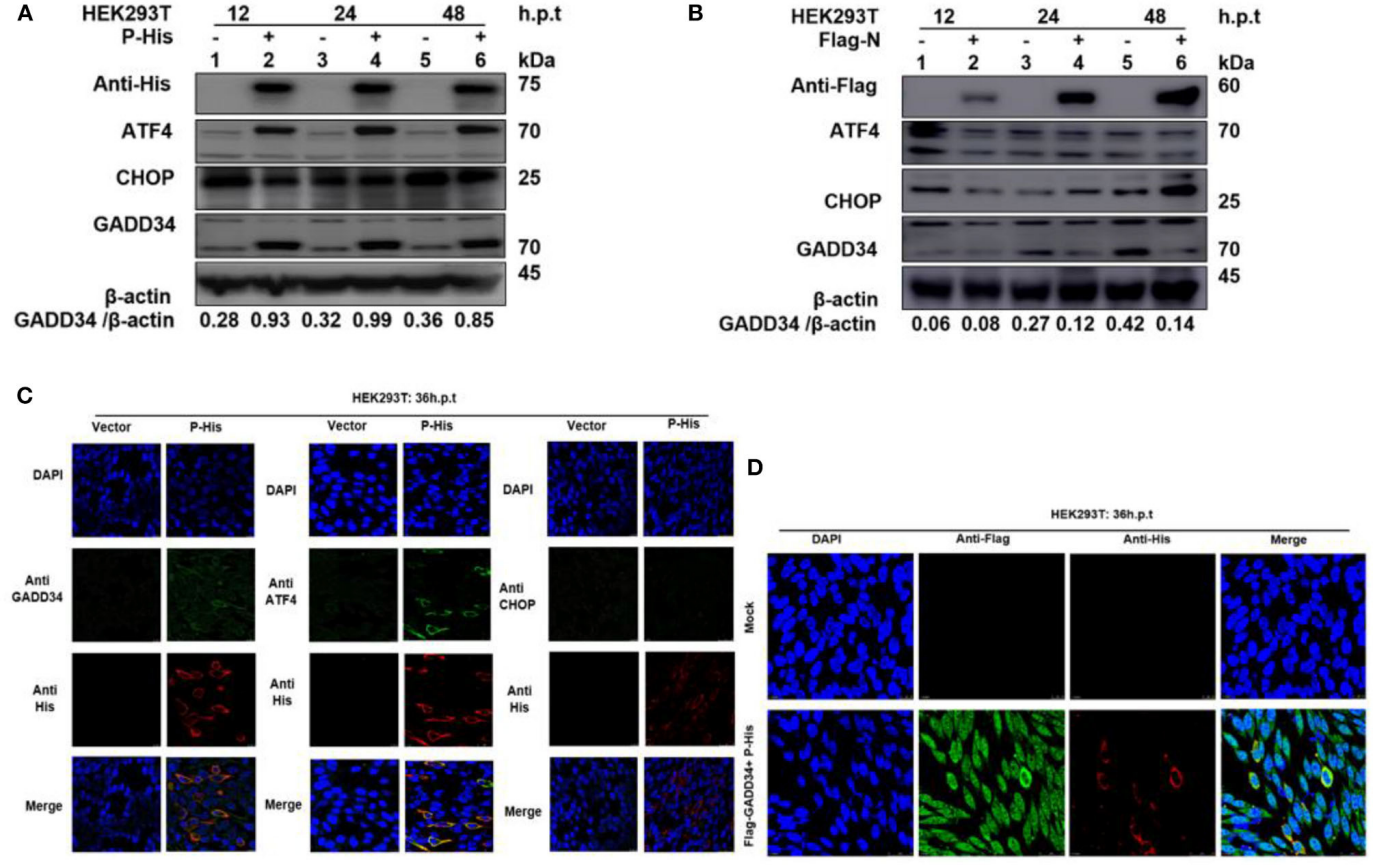
PPRV P protein induces host cellular GADD34. **(A,B)** HEK 293T cells were transfected with either His-tagged P protein expressing plasmid (in **A**) or Flag-tagged N protein expressing plasmid (in **B**) or mock-transfected with an empty vector at indicated times. The effect of PPRV P or N proteins on ATF4, CHOP, and GADD34 protein expression was analyzed by Western blot and β-actin was used as an internal loading protein. **(C)** HEK 293T cells were transfected with His-tagged P protein expressing plasmid or empty vector for 36 h. Cells were fixed and double-stained with Rabbit anti-GADD34 (10449-1-AP) 1:100, Rabbit anti ATF4 (10835-1-AP) 1:100, and Rabbit anti-CHOP (D46F1) 1:50, and His-tagged P protein was stained with Mouse anti-His-tag (66005-1-Ig) 1:200 as primary antibodies overnight. Goat anti-rabbit IgG (H&L) Alexa Fluor 488 (ab150077) Green 1:500 and Goat anti-mouse IgG H&L (Alexa Fluor 647) (ab150115) Red 1:1000 were used as secondary antibodies. DAPI was used to stain the cell nuclei (blue). **(D)** Colocalization of PPRV P protein with GADD34. HEK 293T cells were co-transfected with His-tagged P protein expressing plasmid and Flag-tagged GADD34 for 36 h followed by double staining with Rabbit anti-Flag (ab1162) 1:200 and Mouse anti-His-tag (66005-1-Ig) 1:200 as primary antibodies overnight. Goat anti-rabbit IgG (H&L) Alexa Fluor 488 (ab150077) Green 1:500 and goat anti-mouse IgG H&L (Alexa Fluor 647) (ab150115) Red 1:1000 were used as secondary antibodies. DAPI was used to stain the cell nuclei (blue).

It has been suggested that protein synthesis inhibition is an important protective mechanism against various stresses, including viral infection and ER stress. This immune response is mainly based on the phosphorylation state of eIF2α (37, 38). We have demonstrated the ability of PPRV to dephosphorylate eIF2α and a potential reciprocal interaction between PPRV replication and GADD34 upregulation during infection. Furthermore, we described the potential of PPRV P protein to inhibit PERK/eIF2α phosphorylation. It is known that GADD34 plays a role in pro-apoptotic or pro-survival downstream of the PERK/eIF2α pathway. To preliminarily investigate the probable molecular mechanism by which PPRV P protein blocks eIF2α phosphorylation, the status of the transcriptional related proteins downstream of the PERK/eIF2α pathway was investigated. HEK 293T cells were transfected with His-tagged P protein expressing plasmid or mock-transfected with an empty vector at indicated times. Whole-cell lysates were separated by SDS-PAGE and analyzed by Western blot (Figure 6A) or IFA (Figure 6C) for the protein expression state of transcription factor-4 (ATF4), GADD34, and pro-apoptotic transcription factor CCAT/enhancer-binding protein (CHOP). Results showed that in both Western blot and IFA, GADD34 and ATF4 were highly upregulated in the presence of PPRV P protein compared to vector-transfected cells, whereas the expression of CHOP showed a slight attenuation (Figures 6A,C). Surprisingly, the active form of ATF4 was markedly expressed in PPRV P protein-transfected cells but migrated abnormally on SDS-PAGE in a seemingly spliced manner, resulting in a higher molecular weight (approximately 70 kDa) than expected (50–58 kDa) (Figure 6A). To further investigate whether upregulation of GADD34 and ATF4 proteins under the effect of PPRV P protein was not common to other PPRV structural proteins, another set of HEK 293T cells were transfected (in the same condition) with Flag-tagged N protein or mock-transfected with an empty vector. Cell lysates were analyzed by Western blot probed with specific antibodies to detect GADD34, ATF4, and CHOP. As shown in Figure 6B, it was clear that, unlike PPRV P protein, the PPRV N protein could not activate either GADD34 or ATF4. In contrast, transfection of the individual PPRV N protein slightly decreased the level of GADD34 compared to mock-transfected cells from 24 h.p.t., whereas the ATF4 remained inactive. It is interesting to note that CHOP seems to be upregulated at 48 h.p.t. in PPRV N protein-transfected cells (Figure 6B). Taken together with the conclusion in section Viral P protein is involved in dephosphorylation of eIF2α during PPRV infection, it is reasonable to exclude PPRV N protein from the process of PPRV-mediated GADD34 upregulation and its subsequent eIF2α dephosphorylation. Consider that, in Figures 2C,D, we observed that GADD34 was more concentrated in the nucleus of Vero cells infected with PPRV and that PPRV P protein alone could upregulate GADD34 expression. Hence, we hypothesized that the P protein was involved in the cellular location of GADD34 in cells under PPRV infection. To investigate this, we co-transfected HEK293T cells with plasmids expressing His-tagged P and Flag-tagged GADD34 proteins for 36 h to visualize the location of the two proteins. Cells were fixed and subjected to IFA, as described in Materials and Methods, followed by double staining with anti-His and anti-Flag. The results showed that PPRV P protein and GADD34 were transiently co-expressed. The distribution of GADD34 in the nucleus was enhanced and consistent in co-transfected cells compared to cells transfected with P protein alone, as seen in Figures 6C,D. Interestingly, observed Vero cells transfected with GADD34 prior to PPRV infection showed a similar enhancement of GADD34 distribution in the nucleus and a more pronounced PPRV foci (see Figure 2D), suggesting a reciprocal effect on each other. Altogether, the results in this section indicate that PPRV P protein alone can induce upregulation of host cellular GADD34, thus supporting its involvement in the PPRV-mediated eIF2α dephosphorylation.

## Discussion

**Figure 7:**
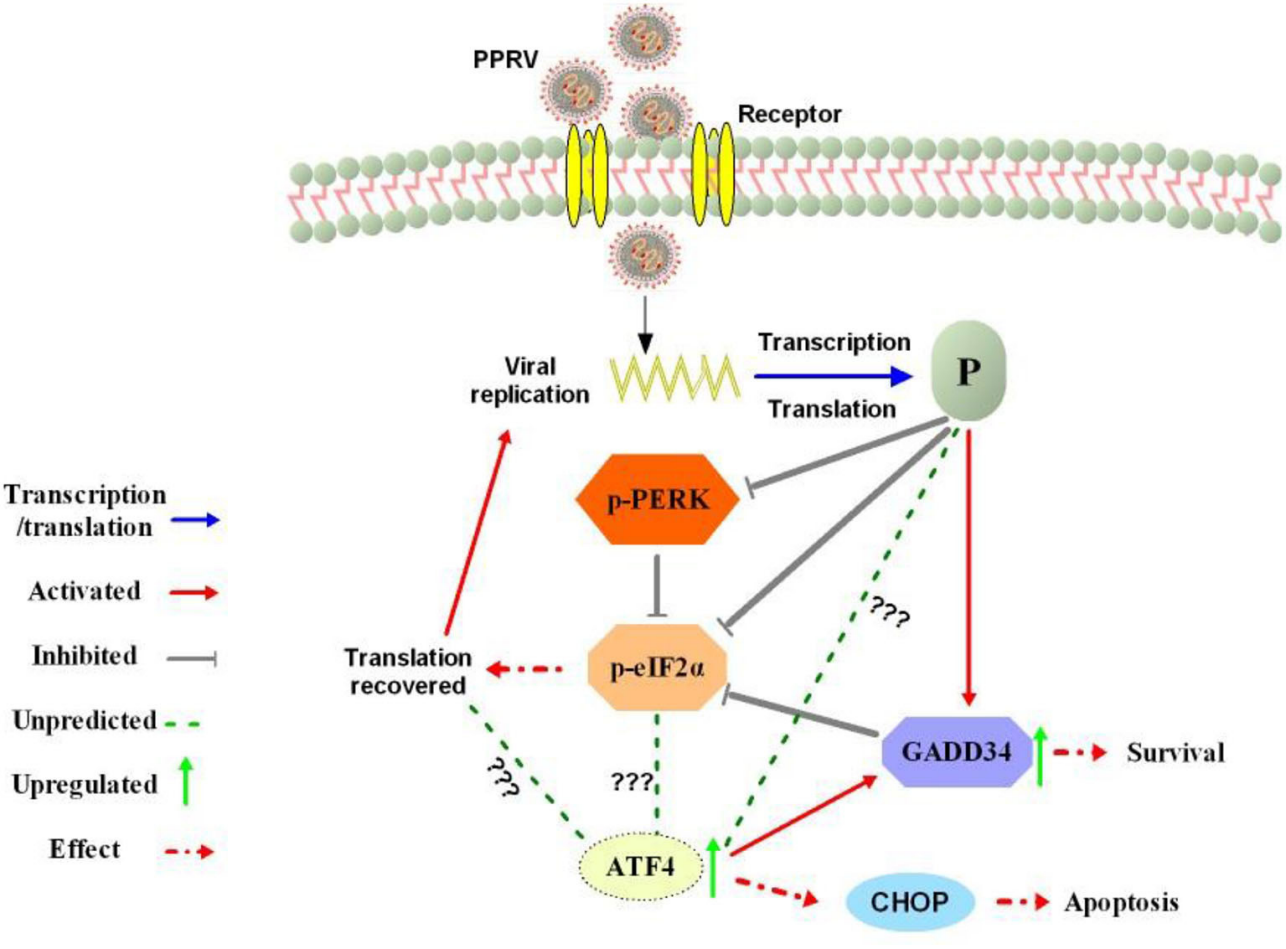
Summary of the predicted modulation of eIF2α phosphorylation by PPRV. After viral entry, the viral phosphoprotein (P) is produced through transcription and translation process. The P protein expression prevents PERK activation and activates cellular GADD34 to block eIF2α phosphorylation through its negative feedback loop that attenuates signaling of integrated stress, thus leading to the cell survival and translation recovery. Translation recovery may then facilitate PPRV replication and viral protein synthesis. The mechanism by which PPRV P protein induces upregulation of ATF4 in the absence of PERK/eIF2α phosphorylation requires further investigation. In fact, ATF4 was associated with cell death or survival decision through a selection of autophagy or apoptosis with or without CHOP expression.

To date, the biology of PPRV and the mechanism of interaction with its hosts is not fully understood. Recently, studies have implicated PPRV V and P proteins in the suppression of STAT-mediated interferon signaling (12, 13). Autophagy and apoptosis have also been described in PPRV-infected cells (16–18). However, knowledge on the molecular mechanisms and viral proteins involved in modulation of cell fate during infection is still lacking (8, 19). In this study, we described the ability of PPRV to dephosphorylate eIF2α in Vero cells: a mechanism that may be important for PPRV to evade host immunity and facilitate viral replication. In previous reports, viruses have been shown to develop strategies to manipulate host translational machinery to facilitate viral replication and host immune evasion (35, 39–41). Several viruses including African swine fever virus (ASFV) (42), herpes simplex virus 1 (43), and Dengue virus (44) have been reported to manipulate host cellular eIF2α phosphorylation to circumvent the host cellular translational responses. In agreement with the aforementioned, our results showed a potential ability of PPRV to dephosphorylate eIF2α. To understand the probable mechanism behind the PPRV-induced repression of eIF2α phosphorylation, we linked our hypothesis with the previous reports on the coronavirus-associated inhibition of PKR and upregulation of GADD34 expression, which played a synergetic role in facilitating coronavirus replication (39) to demonstrate that PPRV-mediated eIF2α dephosphorylation was linked to its induction of cellular GADD34 (Figures 2A–D). In another study, GADD34 was found to associate with the protein phosphatase 1 (PP1) to promote *in vitro* dephosphorylation of eIF2α (25). Moreover, in this study, PPRV infection was shown to repress ER stressors (DTT&TG)-mediated eIF2α phosphorylation, confirming its ability to dephosphorylate eIF2α (Figures 3A–D). It was interesting to observe a slight decrease in PPRV N protein expression following TG treatment as shown in Figure 3C (lines 8–10). Previously, TG treatment had shown a stronger effect on West Nile Virus (WNV) replication, which would be associated with the role of ER calcium stores in virus replication (45). Whether the effect of TG on PPRV replication is similar to that of WNV needs further investigation. In addition, manipulation of eIF2α phosphorylation using Salubrinal and ISRIB (known agonist and antagonist of eIF2α phosphorylation) resulted in a differential regulation of PPRV N protein expression (Figures 4A–F), implying that eIF2α dephosphorylation is directly linked to PPRV replication. Furthermore, we demonstrated that PPRV P protein alone could not only block PERK/eIF2α phosphorylation but also induce GADD34 upregulation. The cellular GADD34 protein is a regulatory subunit of a holophosphatase complex that dephosphorylates eIF2α (25) and has been involved in translational recovery (30, 46). In this study, the detection of a high expression of GADD34 along with a decreased level of p-eIF2α in HEK 293T cells transfected with PPRV P protein is normal and may have some correlation with the previous observation by Brush and Weiser (25) that GADD34 forms a complex with a catalytic subunit of PP1 that leads to the dephosphorylation of eIF2α. Moreover, the induction of cellular GADD34 by PPRV P protein suggests a possible linkage between the blockage of eIF2α phosphorylation and the enhancement of viral replication in Vero cells infected with PPRV. In the canonical pathways, the activation of PERK leads to the phosphorylation of eIF2α, which in turn activates ATF4 and CHOP, leading to global attenuation of translation and protein synthesis that may negatively impact viral protein translation. In contrast, our results did not show any signal of PERK/eIF2α pathway activation, either in PPRV-infected Vero cells (Figure 1) or in PPRV P protein-transfected HEK293T cells (Figure 5). Therefore, it is reasonable to speculate that PPRV inhibition of PERK/eIF2α phosphorylation is one of the viral strategies used by PPRV to circumvent host immunity. The findings by Novoa et al. suggest that despite the normal activity of stress-inducible eIF2α kinases (PERK, GCN2), the levels of phosphorylated eIF2α are markedly lowered in GADD34 overexpressing cells (30). Besides, ATF4-targeted genes are involved in various pathways that promote cell survival and apoptosis (47). It has been demonstrated that long-term survival results in a transcriptional upregulation of transcription factors, such as the ATF4 promoting the expression of adaptive genes (47). Considering the relatively long life cycle of PPRV (20), this virus may have developed a particular mechanism to promote long-term survival of infected host cells to facilitate its replication. Furthermore, induction of GADD34 has been cited in a negative feedback regulatory mechanism that promotes translational recovery, whereas the ATF4 is required for the transactivation of GADD34 promoter in response to ER stress and amino acid deprivation (37). The aforementioned may explain the induction of GADD34 and ATF4 in PPRV P protein-transfected cells in the absence of PERK/eIF2α phosphorylation. Similarly, eIF2α downregulation during viral infection has been reported in other viruses through different pathways. For example, (1) both PKR and eIF2α were downregulated and GADD34 was upregulated in avian infectious bronchitis virus (IBV)-infected Vero, H1299, and HUH7cells (39); (2) induction and subsequent suppression of PERK and eIF2α phosphorylation have been described in dengue virus (44). Consistent with our results, the levels of p-PERK were slightly decreased in both Vero cells infected with PPRV and HEK293T cells transfected with PPRV P protein (Figures 1A,C and 5A,B). Taking a previous conclusion in (30) as reference, we speculate that the blockade of p-eIF2α observed in PPRV P protein-transfected HEK293T and PPRV-infected Vero cells could be linked to the induction of GADD34 by PPRV P protein as demonstrated by Western blot and IFA analysis (Figures 6A,C,D). It was interesting that the effect of both PPRV and PPRV P protein could trigger a stronger distribution of GADD34 in the nucleus of cells overexpressing GADD34 prior to infection or in co-transfection, which gives evidence of an interactive phenomenon between PPRV and cellular GADD34 protein. It seems normal that the activity of GADD34, a growth arrest and DNA damage-inducible protein that functions as a reversing transcriptional inhibition, be enhanced when it is more concentrated in the nucleus. However, the mechanism by which PPRV P protein induces GADD34 upregulation needs further investigation. Previously, the ASFV DP71L protein was implicated in the dephosphorylation of eIF2α and inhibition of CHOP through the recruitment of PP1 (42); the human papillomavirus (HPV) E6 oncoprotein was associated with GADD34 to mediate translational recovery and eIF2α dephosphorylation (38), and the envelope protein E2 of the Hepatitis C virus (HCV) was demonstrated to bind PERK and inhibit the PERK-mediated eIF2α phosphorylation (48). Given the above, we suggest that PPRV could be classified among viruses that exploit eIF2α dephosphorylation pathways to circumvent host cellular translational responses and facilitate its replication. Of note, our results showed that PPRV P protein, not PPRV N protein, could induce GADD34 upregulation, suggesting that PPRV P protein is at least one factor involved in PPRV-mediated eIF2α dephosphorylation. At this level, we could not explain the reason why ATF4 was upregulated in the absence of PERK/eIF2α phosphorylation and why the observed molecular weight of its active form was higher than expected in HEK 293T transfected with PPRV P protein. This observation also suggests a potential interaction (direct/indirect) between PPRV P protein and cellular ATF4 during PPRV infection that requires further investigation. In previous reports (49–52), the ATF4 switch between adaptive and pro-apoptotic gene expression has been attributed to the formation of different ATF4 heterodimers controlling specific targets that follow distinct kinetics of expression. ATF4 has also been described to bind DNA as a homodimer or a heterodimer that is ubiquitinated by stem cell factor (SCF) in response to mammalian target of rapamycin complex 1 (mTORC1) and proteasomal degradation leading to the downregulation of sirtuin4 (SIRT4), presuming that the molecular weight of ATF4 was not stable (53, 54). Furthermore, ATF4 was demonstrated to induce apoptosis in cancer cell lines independently of CHOP (55, 56). The results of these studies suggested a pro-survival role for CHOP, indicating that the transcriptional regulation of ATF4 was more complex than previously thought (47). Besides, ATF4 activates targets involved in autophagy (57) and upregulates targets involved in protein synthesis such as GADD34 to promote eIF2 dephosphorylation and restoration of translation (46). In our case, we estimated that the observed ATF4 product may have undergone one of the aforementioned scenarios in a yet uncharacterized mechanism that requires further investigation. On the other hand, during ER stress, ATF4 is responsible for cell death or survival decision through the selection of autophagy or apoptosis with or without CHOP expression (58). This statement may explain the fact in our case that CHOP expression was attenuated in the presence of a higher expression of ATF4. Moreover, it has been demonstrated that PPRV exploits cellular autophagy machinery for viral replication (16), which may also explain why CHOP expression was attenuated in PPRV P protein-transfected cells, since CHOP is a pro-apoptotic protein. At the protein level, overexpression in mammal cells of individual PPRV P protein showed a high ability of this protein to stimulate cellular GADD34 protein, a property that was absent for PPRV N protein (Figure 6B). Therefore, it is reasonable to conclude that PPRV P protein is in part essential in the regulation of viral/host protein translational machinery through induction of GADD34 and its subsequent eIF2α dephosphorylation, probably leading to cell survival and cellular translational recovery, which is beneficial for PPRV viral protein translation and replication as summarized in Figure 7. Likewise, the ICP34.5 protein of herpes simplex virus was identified to play a role of bridging eIF2α and PP1 during viral replication and anti-host response through eIF2α dephosphorylation (59). Similarly, the nsP4 protein of Chikungunya virus was shown to suppress eIF2α phosphorylation to facilitate replication (60). The basal level of eIF2α phosphorylation was also reduced *via* induction of GADD34 and inhibition of PKR autophosphorylation in cells infected by coronavirus avian IBV (39). In this study, treatment with a selective inhibitor of eIF2α dephosphorylation (Salubrinal) demonstrated evidence of impairment in PPRV replication when eIF2α dephosphorylation is inhibited (Figure 4E). Transfection of PPRV P protein also showed the ability to dephosphorylate eIF2α. Together with previous reports involving PPRV V and P protein in the suppression of STAT-mediated interferon signaling (12, 13), there is a reason to conclude that PPRV P protein is important to PPRV viral immune response and may contribute to host immune evasion during viral infection. Whether the involvement of PPRV P in the induction of GADD34 is the most essential for viral replication and evasion of host immunity remains to be determined. More accurate information should result from the application of reverse genetics techniques to elucidate the exact function of PPRV P protein using recombinants PPRV lacking PPRV P protein or containing a modified PPRV P protein. Unfortunately, this reverse genetics system is less documented and still lacking in the hands of several researchers on PPRV (61) although efforts are being made (62, 63). Another important tool to confirm our observations could be an *in vivo* assay using field virulent strains of PPRV, which would require reference laboratories licensed to handle live PPRV. The lack of suitable animal models for PPRV growth and replication is another burden to carry over more conclusive *in vivo* assays. Overall, the conclusions of our work provide new insights and guidelines for further studies toward the understanding of PPRV pathobiology and its viral/host interactions.

## Data Availability Statement

The raw data supporting the conclusions of this article will be made available by the authors, without undue reservation.

## Author Contributions

NA designed the work, conducted most of the experiments, and drafted the manuscript. BQ assisted in plasmid construction and cell culture while XQ and XY provided technical support and interpretation of data. MP and YL assisted in data analysis and manuscript writing. YD and ZZ were in charge of the funding and the supervision of this work. All authors contributed to the article and approved the submitted version.

## Conflict of Interest

The authors declare that the research was conducted in the absence of any commercial or financial relationships that could be construed as a potential conflict of interest.
